# Integrative analyses of maternal plasma cell-free DNA nucleosome footprint differences reveal chromosomal aneuploidy fetuses gene expression profile

**DOI:** 10.1186/s12967-022-03735-7

**Published:** 2022-11-18

**Authors:** Min Zhang, Kun Li, Shoufang Qu, Zhiwei Guo, Yuanli Wang, Xu Yang, Junhua Zhou, Guojun Ouyang, Rongtao Weng, Fenxia Li, Yingsong Wu, Xuexi Yang

**Affiliations:** 1 Institute of Antibody Engineering, School of Laboratory Medicine and Biotechnology, Southern Medical University, 1838 N. Guangzhou Ave, Guangzhou, 510515 China; 2 National Institutes for Food and Drug Control, Beijing, 100050 China; 3 Guangzhou Darui Biotechnology Co. Ltd., Guangzhou, 510665 Guangdong People’s Republic of China; 4 Department of Obstetrics and Gynecology, Nanfang Hospital, Southern Medical University, Guangzhou, China

**Keywords:** Plasma cell free DNA, Aneuploid fetus, Trisomy pregnancy, Nucleosome footprints, Gene expression profiles

## Abstract

**Background:**

Chromosomal aneuploidy is the most common birth defect. However, the developmental mechanism and gene expression profile of fetuses with chromosomal aneuploidy are relatively unknown, and the maternal immune changes induced by fetal aneuploidy remain unclear. The inability to obtain the placenta multiple times in real-time is a bottleneck in research on aneuploid pregnancies. Plasma cell-free DNA (cfDNA) carries the gene expression profile information of its source cells and may be used to evaluate the development of fetuses with aneuploidy and the immune changes induced in the mother owing to fetal aneuploidy.

**Methods:**

Here, we carried out whole-genome sequencing of the plasma cfDNA of 101 pregnant women carrying a fetus with trisomy (trisomy 21, n = 42; trisomy 18, n = 28; trisomy 13, n = 31) based on non-invasive prenatal testing (NIPT) screening and 140 normal pregnant women to identify differential genes according to the cfDNA nucleosome profile in the region around the transcription start sites (TSSs).

**Results:**

The plasma cfDNA promoter profiles were found to differ between aneuploid and euploid pregnancies. A total of 158 genes with significant differences were identified, of which 43 genes were upregulated and 98 genes were downregulated. Functional enrichment and signaling pathway analysis were performed based on Gene Ontology (GO) and Kyoto Encyclopedia of Genes and Genomes (KEGG) databases found that these signal pathways were mainly related to the coordination of developmental signals during embryonic development, the control of cell growth and development, regulation of neuronal survival, and immune regulation, such as the MAPK, Hippo, TGF-β, and Rap1 signaling pathways, which play important roles in the development of embryonic tissues and organs. Furthermore, based on the results of differential gene analysis, a total of 14 immune-related genes with significant differences from the ImmPort database were collected and analyzed. These significantly different immune genes were mainly associated with the maintenance of embryonic homeostasis and normal development.

**Conclusions:**

These results suggest that the distribution characteristics of cfDNA nucleosomes in maternal plasma can be used to reflect the status of fetal development and changes of the immune responses in trisomic pregnancies. Overall, our findings may provide research ideas for non-invasive detection of the physiological and pathological states of other diseases.

**Supplementary Information:**

The online version contains supplementary material available at 10.1186/s12967-022-03735-7.

## Background

Chromosomal aneuploidy is the most common type of birth defect in fetuses and is the leading cause of various human congenital malformations, such as early miscarriage, perinatal mortality, and severe intellectual disability [1]. NIPT is currently an effective clinical preventive measure that is based on next-generation sequencing (NGS) [2, 3], and has a remarkable contribution to the detection of chromosome aneuploidy, point mutations, and copy number variations [3]. Studies on the developmental mechanism and gene expression profile of fetuses with chromosomal aneuploidy are progressing slowly, and the molecular characteristics of maternal immune changes induced by fetal aneuploidy are unclear. Of note, real-time and repeated acquisition of placental and fetal tissues is a bottleneck in this research area. Several methods have been used to detect the gene expression profile of fetuses with aneuploidy, including DNA microarray analysis, serial analysis of gene expression (SAGE), and the less common quantitative real time polymerase chain reaction (qRT-PCR) [4–7]. Most of these methods require DNA or mRNA samples that are obtained from fetal cells using traumatic methods, such as amniocentesis, umbilical cord puncture, or villus tissue. These operations are associated with risks to the fetus, such as abortion and infection [8], which are of remarkable concerns to pregnant women and their families. Therefore, non-invasive detection methods are needed to obtain the fetal gene expression profiles, identify differential genetic phenotypes in trisomy pregnancy, and determine the potential impact mechanism of the maternal immune response to fetuses.

Plasma cfDNA is mainly derived from rapidly growing and metabolizing cells in the body and carries the basic informative characteristics of its source cells and tissues [9–11]. Plasma cfDNA fragments are released by apoptotic cells after enzymatic chromatin processing. DNA still bound to nucleosomes is retained, whereas naked DNA regions between nucleosomes are digested [12–14], which can be used to infer the tissue of origin of cfDNA [11, 15]. Nucleosomes are depleted in promoters upstream of the TSSs and are well positioned near the TSSs. Nucleosome positioning relative to TSSs is directly correlated with RNA polymerase II (Pol II) binding [16], and genome-wide deep sequencing can be used to identify nucleosome occupancy profiles, which can then be used to simulate the abundance of gene expression [17]. For example, analysis of plasma cfDNA fragments from tumor patients revealed that the promoter regions of active genes exhibited depleted nucleosome coverage, which implied less nucleosome binding in these regions and increased gene expression [17].

During pregnancy, most of the cfDNA in maternal plasma is derived from maternal hematopoietic cells and placental trophoblasts, with only 10% of cell-free DNA derived from the fetus [18, 19]. Many pregnancy complications and fetal developmental defects are rooted in the placenta and involve the maternal immune system [20, 21]. Studies revealed that the distribution characteristics of cfDNA nucleosomes in the plasma of pregnant women vary at different gestational weeks and physiological and pathological states [22]. Accordingly, the use of this difference has been attempted to construct a classifier to predict placental-derived pregnancy syndrome [23, 24]. Simultaneously, the nucleosome footprint of cfDNA has been shown to distinguish breast cancer patients from healthy donors and correlates with the efficacy of neoadjuvant chemotherapy [25]. However, the use of nucleosome footprints of cfDNA to study the mechanisms of aneuploidy fetal in pathology has not been explored. The distribution characteristics of cfDNA nucleosomes carry the gene expression profile information of the cells of their origin. Accordingly, we hypothesized that the information carried by these nucleosomes may be used to study gene expression profiles and functional changes of the immune system in trisomic pregnancies, and thus may provide ideas for the study of other diseases.

Here, we conducted a retrospective study using whole-genome sequencing of plasma cfDNA from 253 participants (42 trisomy 21, 28 trisomy 18, 31 trisomy 13, and 152 normal controls). First, we compared the plasma cfDNA footprint profiles between chromosomal aneuploid samples and healthy controls. Differential genes were further analyzed to identify the biological role via functional annotation and enrichment analyses of GO and KEGG. Our findings indicate that the distribution characteristics of cfDNA nucleosomes in maternal plasma can reflect the gene expression profiles of the placenta and the immune response of pregnant women, and may be used as a potential biomarker of the physiological and pathological states of pregnancy disorders.

## Methods

### Sample collection and study performance

The study was performed using low-throughput sequencing data of 140 healthy pregnant women and the cfDNA library of 101 pregnant women with aneuploid fetuses with trisomy 21, 18, and 13 by Guangzhou Darui Biotechnology Company. The selection of cfDNA sequencing libraries was based on the gestational age at plasma collection and follow-up results for pregnant women with trisomic pregnancy. All selected samples were collected at 12–28 weeks of gestation and the selected participants had singleton pregnancies. The definitive diagnosis of each fetus was confirmed by chromosome testing of the fetus in utero via the collection of amniotic fluid cells, chorionic tissue biopsy, or umbilical cord blood cell culture.

### CfDNA sequencing and data handing

A total of 6 mL peripheral blood was collected in EDTA tubes from each patient and immediately centrifuged at 1600 g for 10 min at 4 °C. The plasma was then transferred to a low-adsorption centrifuge tube (Eppendorf, EP tubes), and re-centrifuged at 16,000 g for 10 min at 4 °C, and the supernatants were collected into new EP tubes and stored at − 20 °C before use. At least 10 ng cfDNA was extracted from plasma for sequencing using the QIAamp DNA Blood Mini Kit (Qiagen). A starting amount of approximately1–5 ng DNA was prepared for library construction. The libraries for sequencing were prepared using the Ion Xpress^™^ Plus Fragment Library Kit and the following steps: end repair, adapter ligation, and amplification with 12 amplification cycles PCR. Libraries were analyzed using a 2100 Bioanalyzer (Agilent Technologies, Singapore) to observe DNA size distribution. Sequencing was performed using the Ion PI^™^ Hi-Q^™^ OT2 200 Kit and Ion PI^™^ Hi-Q^™^ Sequencing 200 Kit.

All samples were sequenced using the Ion Proton platform (Thermo Fisher Scientific, USA). For trisomic pregnancy samples, each cfDNA sample had approximately 20 M sequencing raw data, the sequenced fragment length is 150–160 bp, and the sequencing depth is about 1X. For healthy pregnancy samples, the sequencing raw data of each sample is 5 M, the length of the sequencing fragment is 150–160 bp, and the sequencing depth is about 0.25X. To serve as the control, every 4 low-coverage NIPT data of 140 healthy pregnant women were pooled randomly using “merge” function in SAMtools [26] as the control sample data according to gestational age and a total of 35 bam files were obtained that simulate the high-depth data of healthy pregnant women. Base calling and alignment were performed using Torrent Suite (Version 5.4.0). All reads were mapped to the human reference genome hg19. All results of cfDNA testing and clinical data were transferred to an independent data-coordinating center for consolidation.

### TSS profiles of cfDNA sequencing

Raw bam files processed GC correction using deeptools [27] (Version 3.5.1) to compute GCBias and correct GC bias. The read counts of regions and single base depths ranging from − 1000 bp to + 1000 bp around TSSs were calculated using bedtools coverage [28] (Version 2.17.0) by switching the program argument to “-d”. All gene information and TSS positions were obtained from RefSeq [29]. The read counts were normalized using the reads per kilobase per million mapped reads (RPKM) method.

### Differential TSS analysis

The fold change of each TSS was calculated between groups (N vs. T13, N vs. T18, N vs. T21), and only the TSS with fold change  > 1.5 was used in the Wilcoxon rank sum test (two-sided). The false discovery rate (FDR) method of Benjamini and Hochberg was used for multiple testing corrections. Finally, the TSSs with *p*-value  < 0.05, |log_2_(FoldChange) |≥ log_2_1.5, and FDR  < 0.2 were determined as differential TSSs. Volcano plots were generated using the ggplot2 [30] package, and heatmaps were plotted with pheatmap. All data preparation and screening processes were based on R 4.1.1.

### Gene function analysis

To explore the function of the corresponding genes of differential TSSs, GO and KEGG analyses were performed using the R package, clusterProfiler [31] (Version 4.2.0). The GO terms and KEGG pathways were obtained from the QuickGo [32] and KEGG [33] websites. Immune-related genes were determined using ImmPort database [34]. All gene aliases and ID in the different databases were downloaded from GeneCards [35].

## Results

### Plasma cfDNA of pregnant women with trisomic fetuses and normal pregnant women have different promoter nucleosome distribution characteristics

**Fig. 1:**
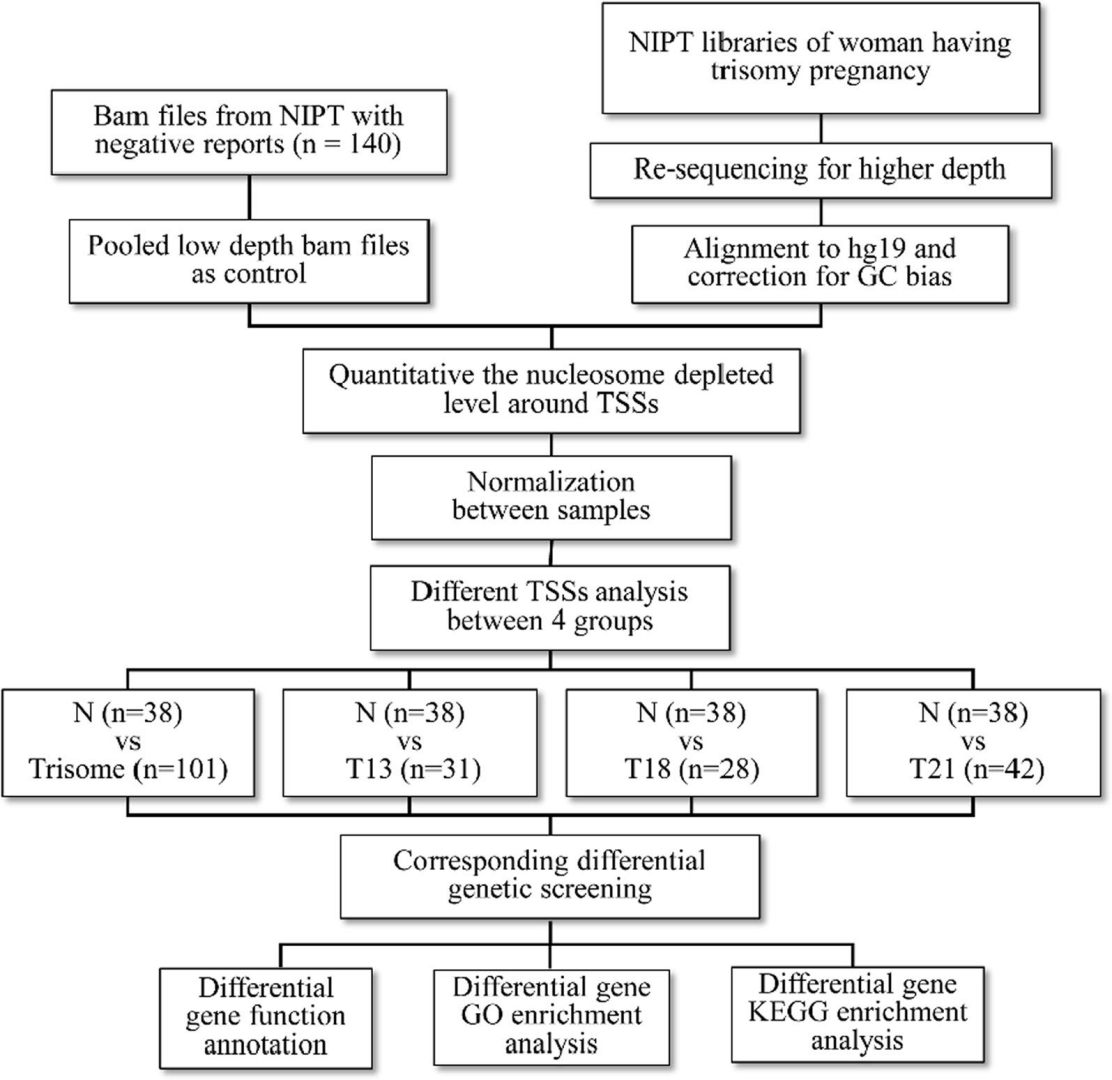
Experimental design and flowchart of data analyses for three trisomic pregnancies study based on cell-free nucleosome footprint low-coverage whole-genome sequencing. The different nucleosome positioning on cfDNA of healthy pregnant women and pregnant women carrying fetuses with trisomy 21, 18 and 13 was analyzed. And finally, the results were integrated analysis and annotated through preprocessing, alignment, quality control, expression index, clustering, and differential analysis of the sequencing data

**Fig. 2:**
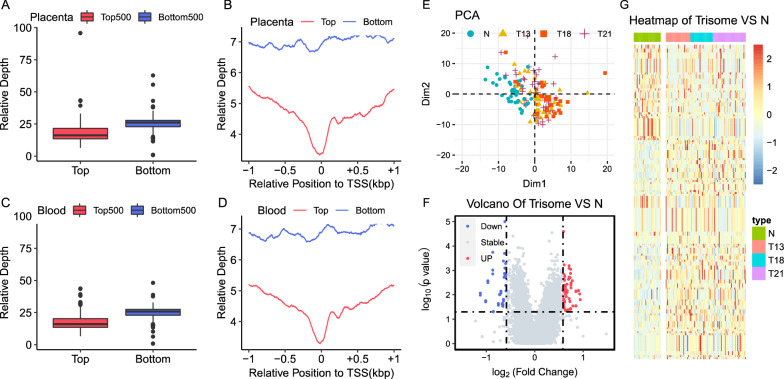
The cfDNA nucleosome footprint in maternal plasma reveals nucleosome positioning and gene expression of maternal and fetal nucleosomes in trisomies. **A** Mean expression levels of the 500 most‐ (Top500, red) and least‐expressed (Bottom500, blue) genes in the placenta. **B** Promoter coverage patterns for the 500 most‐ (Top500, red line) and least‐expressed (Bottom500, blue line) genes in the placenta. **C** Mean expression levels of the 500 most- (Top500, red) and least expressed (Bottom500, blue) genes in the maternal blood. **D** Promoter coverage patterns for the 500 most- (Top500, red line) and least-expressed (Bottom500, blue line) genes in the maternal blood. **E** Principal component analysis (PCA) derived from TSS coverage profiles imputed by different trisomy pregnancies and healthy pregnant women. **F** Volcano plots of gene transcripts with differential read coverages at the pTSS (|log_2_FoldChange|≥ 1.5 and false discovery rate [FDR] < 0.2) at the pTSS detected using whole-genome sequencing for all trisomy pregnancies samples. The blue, red, and gray dots indicate gene promoters though to be downregulated, upregulated, and exhibiting non-differential coverage, respectively. **G** Heatmap of different TSS region coverage between normal pregnancy and trisomic pregnancies

To determine the cfDNA footprint profiles that reflect intracellular nucleosome positioning and predicted gene expression, we selected 101 samples (42 trisomy 21, 28 trisomy 18, and 31 trisomy 13; Additional file 1: Table S1) of the next-generation sequencing library from the Darui Bio-Clinical Examination Center and low coverage sequencing data of cfDNA derived from 140 healthy controls according to the gestational age of plasma collection and their follow-up results (Fig. 1). The gestational age of the healthy controls was matched to the gestational age of each trisomic pregnancy. The study flowchart of the retrospective analysis of whole-genome resequencing of all collected libraries for trisomic pregnancies is shown in Fig. 1. By comparing cfDNA coverage at the primary TSS (pTSS) for the 500 highest and 500 lowest expressed genes in the placenta and maternal blood (Fig. 2A, C), the sequence coverage depth around the TSSs was found to be significantly lower for highly expressed genes than lowly expressed genes in the placenta (Fig. 2B). Similar patterns were evident for genes with high or low expression levels in the maternal blood (Fig. 2D), aligning with the conclusions of some existing studies on the use of nucleosome footprints to identify differential expression genes [22–24]. As the development of trisomy pregnancy is related to the function of the placenta and maternal immune system, these results suggest that the coverage of cfDNA in gene TSS can be used as a biomarker to explore changes in gene expression during trisomy pregnancy. PCA was performed on GC-adjusted TSS profiles, and samples were divided into four groups according to pregnancy status to reflect the differences in TSS profiles between healthy pregnant women and women with trisomy pregnancies (trisomy 21, trisomy 18, and trisomy 13) (Fig. 2E). The resulting score plot showed that trisomy and healthy pregnancies occupied distinct locations in the PCA plot. However, each trisomy was cross-reactive with all other trisomic pregnancies.

We compared the sequence coverage depth around the cfDNA TSSs between pregnant women with trisomy and healthy pregnant women. Among the 50,800 tested TSSs, 158 were significantly different (*p* < 0.05, |log_2_(foldchange)|≥ log_2_1.5 and FDR < 0.2), including 101 TSSs with relatively high coverage and 57 TSSs with relatively low coverage in pregnant women with trisomy (n = 101) compared with healthy pregnant women (n = 38) (Fig. 2F, Additional file 1: Table S1). Hierarchical clustering analyses revealed an obvious separation between pregnant women with trisomy and healthy controls (Fig. 2G). Simultaneously, we compared TSS profiles between pregnant women with different trisomy pregnancies (T21, T18, and T13) and healthy controls. The TSS regions of 242 genes (*p* < 0.05, |log_2_(FoldChange)|≥ log_2_1.5, and FDR < 0.2) were significantly differentially covered in trisomy 21 pregnancies, 73 of which were upregulated and 169 were downregulated (Additional file 6: Fig. S1A; Additional file 1: Table S1). The TSS regions of 517 genes (*p* < 0.05, |log_2_(FoldChange)|≥ log_2_1.5, and FDR < 0.2) were significantly differentially covered in trisomy 18 pregnancies, 157 of which were upregulated and 360 were downregulated (Additional file 6: Fig. S1B, Additional file 1: Table S1). The TSS regions of 293 genes (*p* < 0.05, |log_2_(FoldChange)|≥ log_2_1.5 and FDR < 0.2) were significantly differentially covered in trisomy 13 pregnancies, 105 of which were upregulated and 188 were downregulated (Additional file 6: Fig. S1C; Additional file 1, Table S1). Conversely, the depth of promoter coverage of these genes were not altered in healthy pregnant women. We proceeded to perform unsupervised hierarchical clustering analysis on the coverages for these trisomy syndromes. Distinctive coverage patterns were found for T21 (Additional File 6, Fig. S1D), T18 (Additional file 6: Fig. S1E), and T13 (Additional file 6: Fig. S1F), revealing distinct coverage patterns for T21, T18, and T13. The heatmaps showed distinct patterns of promoter coverage between healthy pregnancies and pregnancies with trisomy (Additional file 6: Fig. S1D-F).

### Gene annotations indicate that differential genes were primarily associated with signaling pathways that lead to abnormal fetal developmental phenotypes in aneuploidy

**Fig. 3:**
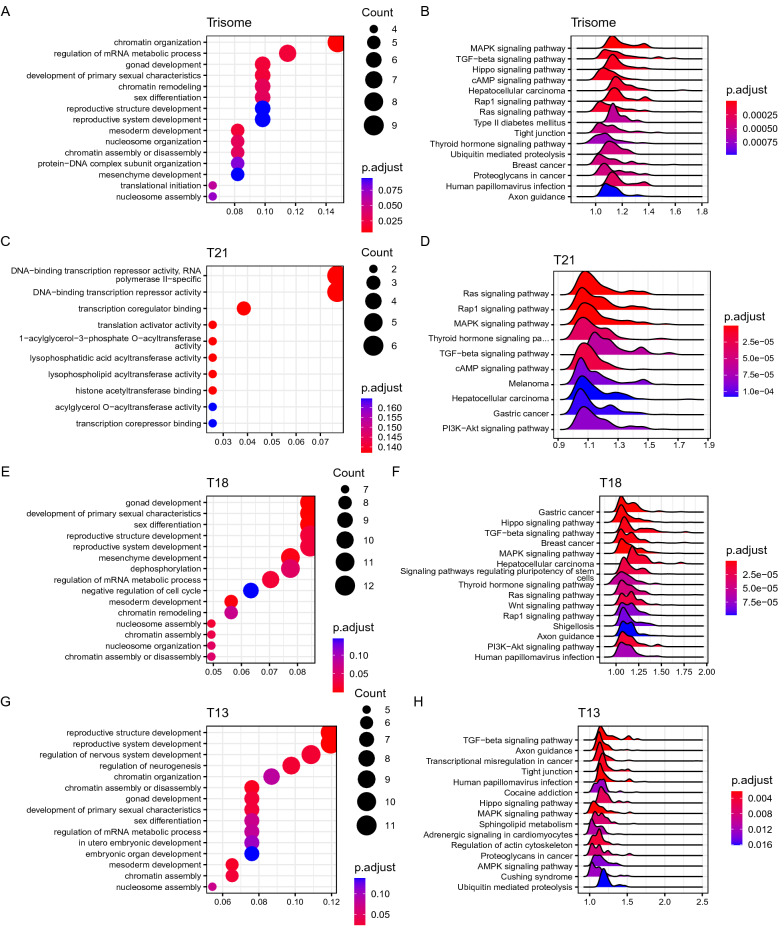
Results of top 15 GO and KEGG analysis results of differentially expressed genes: **A** Bubble chart of GO enrichment analysis in all trisomic pregnancies; **B** Ridgeline plot of KEGG pathway enrichment in all trisomic pregnancies; **C** GO enrichment analysis in trisomy 21 pregnancies; **D** KEGG pathway enrichment in trisomy 21 pregnancies; **E** GO enrichment analysis in trisomy 18 pregnancies; **F** KEGG pathway enrichment in trisomy 18 pregnancies; **G** GO enrichment analysis in trisomy 13 pregnancies; **H** KEGG pathway enrichment in trisomy 13 pregnancies

To study the main functions of the differential genes between pregnant women with trisomy and healthy pregnant women, we performed gene function annotation analysis of the differential genes. Functional annotation analyses revealed that these genes were related to the top 15 terms (Fig. 3A). Trisomy 21, 18, and 13 fetuses commonly have severe mental retardation, prenatal multiple organ malformations, reproductive organ dysplasia, and growth retardation [36–38]. These fetuses result from chromosomal aberrations, the non-disjunction of homologous chromosomes or chromatids that occurs during oocyte meiosis process. Our method enriched many signaling pathways involved in the formation and maintenance of chromatin structures. For example, genes related to chromatin remodeling and nucleosome aggregation (*ARID1A*, *SATB1*, *SETD5*, *EHMT1*, *TSPY8*, and *TSPY10*) were concentrated in the signaling pathways of chromatin formation and structural changes (GO:0,006,333, GO:0,006,334, GO:0,034,728, GO:0,006,338, GO:0,006,325); the related genes are listed in Supplementary Material (Additional file 2: Table S2). Some differential genes were also found to be enriched in pathways related to specific reproductive system development. For example, genes related to gonadal mesoderm development (*TSPY8*, *TSPY4*, *TSPY10*, *TSPY3*, *RBMY1B*, *SF1*) were found to be concentrated in the signaling pathways of gonadal development, reproductive system development, and sex differentiation (GO:0,008,406, GO:0,048,608, GO:0,045,137, GO:0,061,458, GO:0,007,548, GO:0,007,498); the related genes are listed in Additional file 2: Table S2. Diseases that have been reported to be associated with these genes include global stunting with or without mental retardation; autosomal dominant non-syndromic mental retardation; and heart, eye, and genital syndromes [39–41].

We searched the literature to elucidate the functional relevance of the top enriched gene functions and pathways to the diseases. According to the results, each set of enriched pathways was associated with the development of the corresponding disease (Fig. 3B). For example, the Hippo signaling pathway (hsa04390) and TGF-β signaling pathway (hsa04350) play important roles in the regulation of cell growth, differentiation, and development in the human body, which involve different key physiological processes [42, 43]. Based on recent research [44], the Hippo pathway and the TGF-β signaling pathway have a new role in coordinating developmental signals. Hippo signaling plays a key role in early embryonic development, as low Hippo activity is required for trophoblast differentiation whereas high Hippo activity allows inner cell mass formation. Hippo signaling also plays an important role in the embryonic developmental stages of multiple organs, such as the heart, craniofacial development, lungs, eyes, brain, and kidneys [45, 46]. The TGF-β signaling pathway inherently contributes to a wide range of early developmental events, including embryonic patterning, cell fate determination, and dynamic motility [47]. The Ras signaling pathway (hsa04014) controls many cellular responses, such as proliferation, survival, and differentiation. Developmental disorders associated with mutations in the Ras pathway share common phenotypic features, including facial abnormalities, cardiac defects, and impaired growth and development [48]. Ubiquitin-mediated proteolysis pathway (hsa04120) regulate nearly every aspect of cellular events in eukaryotes. Dysregulated ubiquitination can lead to serious consequences and human diseases, including cancer, growth defects, and immune disorders [49, 50]. In addition to these signaling pathways, which play important roles in the overall regulation of embryonic growth and development, we found that these major functional enrichments are concentrated in signaling pathways that regulate embryonic neurodevelopment. The MAPK signaling pathway (hsa04010) is critical for normal development of the nervous system from neural progenitor cells derived from the embryonic mesoderm. Studies have shown that MAPK signaling is important for oligodendrocyte differentiation. Children with reduced levels of MAPK signaling exhibit microcephaly, cognitive impairment, and developmental delay [51, 52]. The axon guidance pathway (hsa04360) is critical for the formation of neuronal networks [53]. The thyroid hormone signaling pathway (hsa04919) affects brain development by regulating gene expression, and is important for neuronal development, and fetal size and survival. Maternal hypothyroidism or deficiency can have severe effects on fetal brain and heart development [54, 55]. Individual genes with significantly different coverages from these pathways are listed in the supplementary material (Additional file 3: Table S3). These signaling pathways were found to display significant differential enrichment in trisomy pregnancies.

In addition to the common differential analysis of these chromosomal aneuploid pregnancies, we also performed functional enrichment analysis of differential genes between cfDNA nucleosome footprints of healthy pregnant women and pregnant women carrying a fetus with trisomies 21, 18, and 13, respectively. Although these differentially gene-enriched pathways were not significantly different among the three trisomic pregnancies, the number of differentially enriched genes in the pathways was found to differ in different trisomic pregnancies (Fig. 3C–H). In trisomy 18 pregnancies, the differential genes were mainly enriched in reproductive system and mesoderm development (GO:0,008,406, GO:0,048,608, GO:0,061,458, GO:0,007,498, GO:0,045,137, GO:0,060,485) (Fig. 3E). Interestingly, the number of enriched differential genes in trisomy 18 pregnancies was significantly higher than the number of differential genes in the same signaling pathway in the other two aneuploid pregnancies; the related genes are listed in Supplementary Material (Additional file 2: Tables S2, Additional file 3: Tables S3). In trisomy 13 pregnancies, differential genes were enriched for multiple biological processes, from embryo implantation to embryonic organ development, during embryonic development in utero. These pathways, which play important roles in the regulation of embryonic development (GO:0,007,566, GO:0,016,331, GO:0,048,568, GO:0,001,838, GO:0,048,568, GO:0,001,701) (Fig. 3G), were not significantly enriched in the other two aneuploid pregnancies. Compared to other children with chromosomal aneuploidy, children with trisomy 18 have obvious genitourinary abnormalities and abnormal developmental delays. Fetuses with trisomy 13 have fewer stable embryos. The up- and down-regulation functions and pathways of differential gene enrichment in the three trisomic pregnancies are presented in the Supplementary Material (Additional file 6: Figs. S2, S3), and the related genes are listed in the Supplementary Material (Additional file 6: 3–5).

According to the literatures, survivors of common trisomy aneuploidies have obvious growth and developmental disorders and mental retardation. Our differential gene enrichment results precisely focused on the pathways of cell growth and development and neurodevelopmental regulation, which correspond well with the shared genetic phenotypes of fetuses with trisomy. In addition, our differential genes in different chromosomal aneuploid pregnancies were focused on their respective and most characteristic genetic phenotypes, indicating that our method may reveal good evidence related to fetal gene expression profiles in trisomic pregnancy.

### Gene annotation indicates that immune-related differential genes may be primarily associated with maternal immune responses to fetal developmental abnormalities

**Fig. 4:**
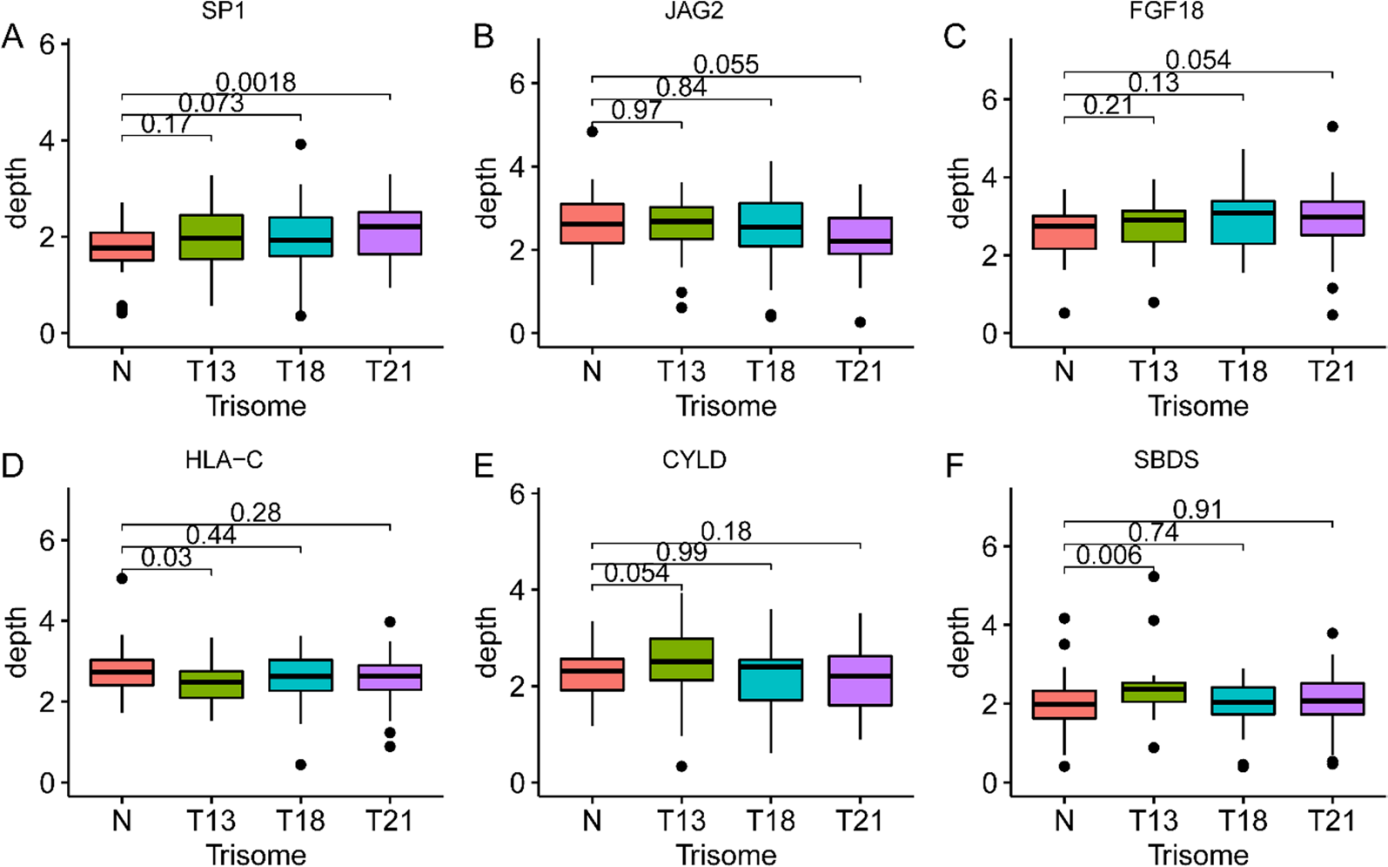
Dysregulated immune genes detected in maternal serum in trisomic pregnancies. Cell-free DNA nucleosome coverage (Z-scored original value) of six selected immune genes with significant difference between healthy pregnancies and different trisomic pregnancies. The extremes of the boxes define the upper and lower quartiles, and the center lines define the median. Whiskers indicate 1.5 times the interquartile range (IQR). The value on the boxplot was the depth of these immune genes in the TSS region of the cfDNA of different trisomic pregnancies, respectively

To determine whether these differential genes were associated with some immune responses in trisomic fetuses during pregnancy, all genes with significantly differential TSS obtained from trisomic pregnancy samples that were screened out were entered into the immune gene database, ImmPort database [34], for comparison and search for the immune genes among these significantly different genes by generating intersections. Fourteen distinct immune genes were identified, and the boxplot shown the relative depth of the region around the TSS of some immune-related genes in the cfDNA of pregnancies in different groups (Fig. 4A–F). Among them, trisomy 13 pregnancy had six distinct genes, trisomy 18 pregnancy had seven distinct genes, and trisomy 21 pregnancy had three distinct genes. We searched the literature for correlations between different immune genes and fetal and maternal immune responses. According to our findings, these significantly different immune genes are related to cell differentiation, growth arrest, and apoptosis, including embryogenesis and tissue homeostasis. The expression of these immune genes, especially the differential genes that play important roles in embryogenesis, differentiation, and development, was significantly different in the promoter profiling of different trisomic pregnancies and healthy pregnant women (Fig. 4A–F).

For example, *Sp1* (*Sp1* Transcription Factor) has an important role in the development of blood cells. Loss of *Sp1* results in general dysregulation of the timing and coordination of differentiation trajectories during hematopoietic specification, such as uncoordinated and unstable embryonic development [56]. *FGF18* (Fibroblast Growth Factor 18) is expressed in embryo and is required for osteogenesis and chondrogenesis. Studies have shown that deletion or mutation of *FGF18* results in reduced proliferation of calvarial osteogenic mesenchymal cells during embryonic development, and terminal differentiation to calvarial osteoblasts is specifically delayed [57]. *CYLD* (Deubiquitinating Enzyme) helps regulate cell survival, proliferation, and differentiation through its effect on *NF-κB* activation. *CYLD* is important for the TNF signaling pathway, and a lack of the *CYLD* gene leads to early embryonic lethality [58]. *HLA-C* (Human Leucocyte Antigen-C) is the only classical MHC molecule expressed by extravillous trophoblasts (EVTs). During pregnancy, *HLA-C* mediates the interaction of EVT with maternal KIR and regulates trophoblast invasion necessary for placentation and overall fetal growth, which in turn affects placental formation and pregnancy outcomes [59, 60]. The Notch signaling pathway is essential for proper embryonic development, and mutations in its components disrupt embryonic development and cause inherited diseases in humans. The Jagged2 (*JAG2*)-mediated Notch signaling is essential for proper craniofacial morphogenesis and proper thymic and limb development in embryos [61]. *SBDS* (*SBDS* ribosomal maturation factor) is an essential gene for early mammalian development, and its expression pattern is consistent with its critical role in cell proliferation. Deletion of *SBDS* results in early embryonic lethality and ectoderm development failure [62]. All related genes are listed in the Supplementary Material (Additional file 1: Table S1).

Interestingly, the expression of these immune genes, which play important roles in regulating cell growth and development, embryonic differentiation, and maturation in our analysis, is consistent with those reported in previous studies. In the early and second trimesters of pregnancy, the fetal DNA population only accounts for approximately 10% to 20% of the maternal plasma free DNA, called the fetal part [19, 63]; the remaining population is mainly maternal DNA. Since the cfDNA of mother was mainly derived from immune cells, these results suggested that these immune genes may be associated with the mother’s immune response to the fetus.

## Discussion and conclusions

Previous studies on chromosomal aneuploidy screening based on maternal plasma cfDNA focused on expanding the non-invasive prenatal detection of trisomy 21, 18, and 13 to all chromosomes by increasing the amount of high-throughput sequencing data and microdeletion/microduplication (expanded non-invasive prenatal test, non-invasive prenatal test PLUS, and NIPT PLUS) [64]. Studies to determine how all or part of the genes involved in chromosomal aneuploidy lead to the genetic phenotype of trisomic fetuses are progressing slowly. Whether the mother's immune system in a trisomic pregnancy produces a series of immune responses to the fetus remains unclear. Centralized methods can use DNA or mRNA samples from different cells, tissues, or whole organs to study gene expression profiles. However, these techniques rely on the use of traumatic methods to obtain fetal cells or villi, and these operations have a certain risk of miscarriage and ethical requirements. In NIPT-related research based on NGS, the distribution characteristics of plasma cfDNA nucleosomes were found to significantly differ in placental trophoblast cells and maternal hematopoietic cells, and can be used to quantify the proportion of cfDNA in plasma cfDNA [65]. Further, the distribution characteristics of plasma cfDNA nucleosomes can be identified by whole-genome deep sequencing technology. According to recent studies, the distribution characteristics of cfDNA nucleosomes carry the gene expression profile information of their source cells, which can be used as an evaluation index for the pathological and physiological status of the mother and fetus, can be used earlier in placental pregnancy syndrome. Therefore, we propose the use of cfDNA nucleosome imprinting to explore the mechanisms of fetal gene expression profiles and maternal immune responses in trisomic pregnancies.

In this study, we used promoter profiling of whole-genome sequencing of cfDNA from pregnant women to assess the transcriptional activity of cfDNA in its tissues of origin. Differential read depths at the pTSS indicated differential gene expression in the tissues of origin (Fig. 2). Therefore, we hypothesized that the differential read-depth patterns of cfDNA at promoters carry information on fetal and maternal-related diseases. Our findings suggested that differential gene enrichment results based on cfDNA nucleosome imprinting analysis were well associated with the genetic phenotypes of trisomic fetuses, such as mental retardation, growth and development disorders, and multiple malformations. Based on these differences, we separately analyzed the differential functions or pathways found in the three aneuploid fetuses. Interestingly, in addition to the common differences in each trisomic pregnancy, the differential genes for each aneuploidy enriched in their unique genetic phenotype. For example, in trisomy 21 pregnancies, differential genes were mainly enriched in pathways related to neurodevelopment and infection. This finding is consistent with the genetic characteristics of fetal trisomy 21, which has obvious intellectual backwardness and low immune function and is susceptible to various infections. In trisomy 18 pregnancies, the differential genes were mainly enriched in the functions of reproductive structure development and mesoderm development, which is highly consistent with genitourinary system abnormalities and delayed developmental abnormalities in fetal trisomy 18. In trisomy 13 pregnancies, differential genes were enriched in multiple signaling pathways related to the regulation of embryonic development, which is consistent with the genetic characteristics of fetal trisomy 13, including fewer stable embryos, being more prone to miscarriage, and having more severe lethal malformations than the first two types of trisomic fetuses.

CfDNA in plasma is derived from apoptotic cells, including cfDNA released by maternal cells and fetuses. In the plasma of pregnant women, most cfDNA is derived from maternal immune cells, with only approximately 10% of the total cfDNA from the placenta [18]. Therefore, genetic changes based on cfDNA analysis not only reflect those of the fetuses, but also the mothers, especially the immune system. We also identified the differential immune genes based on a comparison of the immune gene library and found that these significantly different immune genes are mainly involved in the immune process that regulates cell survival, proliferation, differentiation and migration, embryogenesis, and tissue stabilization. Interestingly, the expression levels of these genes were significantly downregulated. In general textbooks, the concept of a non-responsive fetus remains prevalent. However, according to recent studies, in gestational week 13, human fetuses develop a more unusual immune system than previously thought [66]. This system can recognize foreign proteins but is not as prone to attack as a mature immune system. Most of the NIPT samples were collected from the week 12 to week 15 of gestation. Therefore, we speculate that these significantly different immune genes may be a series of immune responses to fetal trisomy after the mother recognizes an abnormal pregnancy, which leads to the spontaneous abortion of most trisomic pregnancies during pregnancy.

In summary, using cfDNA nucleosome footprint, we found that some existing and undiscovered genetic differences in trisomy 21, 18, and 13 pregnancies may lead to disease phenotypes. Cell-free DNA in maternal plasma is considered to be DNA of mixed origin. Although fetal and maternal cell-free DNA differs in various aspects, such as quantities, abundance, and fragment size [67], maternal and fetal cell-free DNA cannot be differentiated by sequencing cell-free DNA in maternal plasma alone. Therefore, GO and KEGG analyses could not determine whether the differences in these genes were from the mother or the fetus. Differentiation of fetal and maternal cell-free DNA from maternal blood samples may require the use of epigenetic markers (e.g., methylation) or polymorphic loci. Most studies have shown that changes of development-related functions and pathways may be related to the fetus, while whether the other changes belong to the mother or the fetus remains unclear. Of note, our analyses are considered exploratory. With the accumulation of more NIPT data and larger-scale studies to verify our findings, this method may be used to dynamically monitor changes in fetal and maternal gene expression profiles in aneuploid pregnancies to examine these genetic diseases for a long time and achieve eugenics. Overall, this method may not only be used to explore and predict the occurrence of other neonatal genetic diseases, but also provide new ideas for disease prediction, diagnosis, or treatment.

## Supplementary Information

**Additional file 1:** **Table S1.** Differential promoter coverage genes and GO, KEGG enrichment analysis results of differential genes for all pregnant women with trisomy.

**Additional file 2:** **Table S2.** Differential promoter coverage genes and GO, KEGG enrichment analysis results of differential genes for pregnant women with trisomy 21.

**Additional file 3:** **Table S3.** Differential promoter coverage genes and GO, KEGG enrichment analysis results of differential genes for pregnant women with trisomy 18.

**Additional file 4:** **Table S4.** Differential promoter coverage genes and GO, KEGG enrichment analysis results of differential genes for pregnant women with trisomy 13.

**Additional file 5:** **Table S5.** Immunity genes with differential promoter coverage in all trisomic pregnant women.

**Additional file 6:** All additional figures.

## Data Availability

All datasets generated for this study are included in the article/supplementary material.
