# Supplementary material for: Integrative analyses of maternal plasma cell-free DNA nucleosome footprint differences reveal chromosomal aneuploidy fetuses gene expression profile
Source: J Transl Med. 2022 Nov 18;20:536. doi: 10.1186/s12967-022-03735-7 (PMC9673457; doi:10.1186/s12967-022-03735-7)
Supplement: Supplementary file 6 — Additional file 6: All additional figures. [file 12967_2022_3735_MOESM6_ESM.docx]

***Supplementary Material***

# Supplementary Figures

**Supplementary Figure 1.** Gene transcripts with differential read coverages at primary TSS (pTSS). Volcano plots of gene transcripts with differential read coverages at the pTSS (|log2FoldChange|≥ 1.5 and false discovery rate [FDR] < 0.2) at the pTSS detected using whole-genome sequencing for (A) T21, (B) T18, and (C) T13. The blue, red, and gray dots indicate gene promoters though to be downregulated, upregulated, and exhibiting non-differential coverage, respectively. Heat map of the z-scores of promoters with differential read coverages for (D) T21, (E) T18, and (F) T13.

**Supplement Figure 2.** Bubble charts of top 10 GO enrichment analysis results of differentially expressed genes: (A) Up-regulated terms of GO enrichment analysis in trisomy 21 pregnancies; (B) Down-regulated terms of GO enrichment analysis in trisomy 21 pregnancies; (C) Up-regulated terms of GO enrichment analysis in trisomy 18 pregnancies; (D) Down-regulated terms of GO enrichment analysis in trisomy 18 pregnancies; (E) Up-regulated terms of GO enrichment analysis in trisomy 13 pregnancies; (F) Down-regulated terms of GO enrichment analysis in trisomy 13 pregnancies. The y-axis represents GO-enriched terms. The x-axis represents the fold of enrichment. The size of each circle indicates gene count. The color of the dots represents the adjusted *P*-value. GO, Gene Ontology.

**Supplement Figure 3.** The top 10 KEGG enrichment analysis results of differentially expressed genes: (A) Up-regulated terms of KEGG enrichment analysis in trisomy 21 pregnancies; (B) Down-regulated terms of KEGG enrichment analysis in trisomy 21 pregnancies; (C) Up-regulated terms of KEGG enrichment analysis in trisomy 18 pregnancies; (D) Down-regulated terms of KEGG enrichment analysis in trisomy 18 pregnancies; (E) Up-regulated terms of KEGG enrichment analysis in trisomy 13 pregnancies; (F) Down-regulated terms of KEGG enrichment analysis in trisomy 13 pregnancies. KEGG, Kyoto Encyclopedia of Genes and Genomes.
